# Targeted delivery of autoantigen to dendritic cells prevents development of spontaneous uveitis

**DOI:** 10.3389/fimmu.2023.1227633

**Published:** 2023-09-01

**Authors:** Izabela P. Klaska, Tian Yu, Rosie Fordyce, Koju Kamoi, Richard J. Cornall, Cristina Martin-Granados, Lucia Kuffova, John V. Forrester

**Affiliations:** ^1^ Institute of Medical Sciences, University of Aberdeen, Aberdeen, United Kingdom; ^2^ Department of Ophthalmology, Beijing Hospital, National Center of Gerontology, Beijing, China; ^3^ Department of Ophthalmology and Visual Science, Graduate School of Medical and Dental Sciences, Tokyo Medical and Dental University, Tokyo, Japan; ^4^ Nuffield Department of Medicine, University of Oxford, Oxford, United Kingdom; ^5^ Eye Clinic, Aberdeen Royal Infirmary, Aberdeen, United Kingdom

**Keywords:** DEC205, dendritic cells, fusion protein, autoimmune, uveitis, hen egg lysozyme

## Abstract

Restoration of immunological tolerance to self antigens has been a major drive in understanding the mechanisms of, and developing new treatments for, autoimmune and autoinflammatory disease. Sessile dendritic cells (DC) are considered the main instruments underpinning immunological tolerance particularly the CD205^+^ (DEC205^+^) cDC1 subset in contrast to DCIR2^+^ cDC2 which mediate immunogenicity. Targeting DC using autoantigen peptide-antibody fusion proteins has been a well explored methodology for inducing tolerance. Here we show that subcutaneous (s.c.) inoculation of hen-egg lysozyme (HEL)-DEC205 Ig fusion prevents the development of spontaneous uveoretinitis (experimental autoimmune uveoretinitis, EAU) in a transgenic mouse model generated by crossing interphotoreceptor retinol binding protein (IRBP)-HEL (sTg HEL) with HEL specific TCR (sTg TCR) mice. Prolonged suppression of EAU required injections of HEL-DEC205 Ig once weekly, reflecting the half life of s.c. DC. Interestingly, HEL-DCIR2 Ig also had a suppressive effect on development of EAU but less so than DEC205 Ig while it had minimal effect on preventing the retinal atrophy associated with EAU. In addition, HEL-DEC205 Ig was only effective when administered s.c. rather than systemically and had no effect on EAU induced by adoptive transfer of HEL-activated T cells. These data demonstrate the importance of systemic (lymph node) rather than local (eye) antigen presentation in the development of EAU as well as suggest a potential therapeutic approach to controlling sight-threatening immune-mediated uveitis provided relevant antigen(s) can be identified.

## Introduction

Uveitis is a major source of visual impairment accounting for up to 10% of all causes of blindness (1, 2). Infectious agents account for a significant proportion of cases but in a majority of patients no specific cause can be identified (3). Such cases are currently described as “undifferentiated” uveitis (4) although latent or persistent infection may be lurking undetected (5). Autoimmune/autoinflammatory processes are considered to play a role in many cases of undifferentiated uveitis, concepts based on experimental models of uveitis induced by retina-specific antigens (6). This has been supported by the generation of a transgenic T cell receptor (TCR) mouse model of spontaneous experimental autoimmune uveitis/uveoretinitis (EAU), in which ~30% of pathogenic T cells are specific for the retinal protein interphotoreceptor retinol binding protein (IRBP), underscoring a role for an autoimmune pathogenesis in this disease (7).

We have developed a model of spontaneous EAU, in which the neoantigen hen egg lysozyme (HEL) is expressed in the retina under control of the IRBP promoter (8). When single transgenic IRBP-HEL mice (sTg HEL mice) are crossed to mice expressing a transgenic T cell receptor (TCR) for HEL (sTg TCR mice), double transgenic mice (dTg IRBP : HEL TCR mice, hereafter termed dTg mice) spontaneously develop EAU with onset around post-natal day (P)20/21 and with 100% incidence. Inflammation progressively worsens to reach a peak at ~P30 and eventually leads to total retinal destruction, with phthisis bulbi (ocular shrinkage) occurring in some mice (9). The disease thus resembles the most severe forms of chronic, progressive undifferentiated uveitis in humans (10, 11).

Restoration of self-tolerance is the central therapeutic aim in autoimmune disease. In health, steady-state dendritic cells (DC) promote tolerance through a variety of mechanisms including deletion and anergy of autoreactive T cells but predominantly by maintaining the peripheral pool of T regulatory cells (Tregs) [reviewed in refs (12, 13)]. Since DC’s were shown to have specific cell surface molecules which reflected their functional state, attempts were made to modify the role of DC either for immunogenicity (DC vaccine) or for tolerance induction (14). Initially DEC205, a C-type lectin endocytic receptor, was considered to promote tolerance while delivery of antigens via a second molecule, DC inhibitory receptor 2 (DCIR2), was considered immunogenic and therefore more suitable to induce immune responses against pathogens and tumours. However, these distinctions became blurred as both molecules as well as other surface receptors, were found to have differential effects depending on the microenvironmental context.

Recent developments in DC biology and classification show that homeostatic immunological self-tolerance is sustained particularly by a subset of conventional DC (cDC1) which express CD11c, CXCR1 and DEC205 surface receptors [reviewed in ref (15)]. DEC205, has been shown to mediate tolerogenic properties of DC in the absence of micro-environmental challenge by acting as a scavenging receptor for apoptotic and necrotic cells (16, 17). However, DEC205^+^ DC may also participate in immunogenic/inflammatory responses indicating that the context of antigen uptake and presentation is important in determining the final immune outcome e.g. to foreign antigens (18).

DEC205 is one of a set of DC surface molecules including DCIR2, Langerin, CD11b, CD11c, CD47 and CD40 which have been used as fusion proteins to deliver antigens to DC. While most of these proteins have been delivered with aim of inducing enhanced immune responses in vaccination protocols e.g. against HIV and Leishmaniasis (19, 20), the use of DEC205 antibody, or short chain antibody fragments, fused to “self” antigens, in the absence of adjuvant or other proinflammatory stimuli, has been investigated for its potential to induce antigen-specific immunological tolerance for more than 15 years [reviewed in ref (21)]. Preclinical examples include models of diabetes (22), experimental autoimmune encephalomyelitis (23), experimental colitis (24), and arthritis (25). However, previous studies have indicated that inhibition using chimeric anti-DEC205 might not be sufficient to inhibit ongoing autoimmune responses (26) or chronic disease (27) (**Table 1**).

**Table 1:** Induction of tolerance vs immunity by targeting DC surface molecules.

Published report	DC surface antigen	Disease/Process	Target Antigen	Effect	reference
Ng et. al.	DEC205	HIV	Gag	immunogenic	(19)
Matos et. al.	DEC205	Leishmania	LMSTi	immunogenic	(20)
Price et. al.	DCIR2	diabetes	β cell antigen	tolerogenic	(27)
Cao et. al.	DEC205	apoptotic cells	keratin	immunogenic/tolerogenic	(28) (29)
Tabansky et. al.	DCIR2	encephalo-myelitis (EAE)	proteolipid protein	tolerogenic	(30)

Reuter et al. (31) have specifically investigated the kinetics of antigen uptake using a range of antigen-antibody fusion proteins. They have shown that DEC205 is most efficiently targeted for antigen delivery in steady state cDC1, CD8α^+^DC. In contrast, in activated cDC1, CD8α^+^DC, and in mature cDC2, CD8α^-^DC where DEC 205 is upregulated, antigen-delivery is 50% less efficient (32). Thus the nature of DC and its surface receptor as well as the context in which it is targeted by an antigen-antibody fusion protein determine the immunological outcomes of tolerogenicity vs immunogenicity.

We have tested fusion proteins which combine HEL with antibodies to DC surface molecules, namely DEC205 (cDC1) and DCIR2 (cDC2) in dTg mice prior to the onset of EAU (P18). We show that weekly subcutaneous (s.c.) administration of the DEC205-HEL fusion protein completely abrogates development of EAU while DCIR2-HEL fusion protein is less effective but still significantly inhibitory. Both the inflammatory changes and the associated retinal degeneration (chorioretinal atrophy) are markedly reduced by treatment with DEC205-HEL fusion protein while DCIR2-HEL fusion protein is less effective in suppressing both inflammation and atrophy.

## Materials and methods

### Animals and EAU model

B10BR (H-2^K^) wild type and Tg mice were bred in established breeding colonies and housed in the Medical Research Facility, University of Aberdeen. Littermate male and female mice of different ages were used in the experiments. Generation of dTg mice was as described previously (8). sTg HEL mice expressing HEL antigen under the retina-specific IRBP promoter were crossed with sTg TCR (3A9) mice which have >70% HEL-specific peripheral CD4^+^ T cells. The dTg offspring spontaneously develop EAU at P20/21 with 100% incidence. Genotyping of the experimental mice was performed using standard in-house PCR procedures (9). Appropriate genotype and age matched mice were used in experiments as specified. All animal work was performed in accordance with guidelines of the Association for Research in Vision and Ophthalmology (ARVO) statement for the use of animals in ophthalmic and vision research and the regulations of the Animal License Act 2006 (amended 2019) (United Kingdom).

### Preparation of fusion proteins

Plasmids containing the DNA constructs for DEC205-HEL, DCIR2-HEL, DEC205-OVA and DEC2O5-IgG isotype were obtained from R.M. Steinman and Michel Nussenzweig, Rockefeller University, NY, USA. Fusion proteins were prepared according to the protocol described previously (33). Briefly, both heavy chain and light chain antibody DNA was transfected into HEK293T cells with calcium-phosphate. Cells were grown in complete DMEM and incubated overnight until the next morning when cells were washed with PBS and cultured in complete DMEM for 4 days. Medium containing the hybrid antibodies was then spun down, filtered and kept on ice. Antibodies were purified on Protein G columns (Genescript, Piscataway, NJ, USA; cat. number L00209), and concentrated using Amicon Ultra-15 Centrifugal filter units (10K) (Millipore, UK; cat. number UFC9 010 008). The concentrations of purified antibodies were determined using Nanodrop, and Ig heavy chains fused to HEL peptide were verified by Western blotting before being injected into mice. Rabbit anti-mouse polyclonal antibodies were used for immunoblotting: anti-ovalbumin antibody (Abcam, UK; cat. number ab181688, 1:500 dilution), anti-lysozyme antibody (Rockland, CA, USA; cat number USA 200-401-072, 1:500 dilution) and secondary antibody Alexa Fluor 680 goat anti-rabbit IgG (H+L) (Invitrogen, UK 1:10000 dilution). The membranes were imaged with Odyssey Infrared Imaging System (Licor Biosciences, UK).

### Treatment of dTg mice with fusion proteins

Fusion proteins (DEC205-HEL or DCIR2-HEL; 5µg) were inoculated into dTg mice s.c. into the nape of the neck either as a single dose on P18, or in 6 doses given in weekly intervals (P18, P25, P32, P39, P46, P53), or to assess treatment of ongoing disease in 5 doses (P25, P32, P39, P46, P53). Additional control groups included PBS injection, Ig isotype and DEC205-OVA fusion protein. The development and severity of uveitis was monitored using an otoscope-based fundus imaging system (see below) at the following time points: P21, P30, P45, and P60. For some experiments mice were inoculated with 7 doses of fusion protein (P18, P25, P32, P39, P46, P53, P60) and were examined at P50, P70, P85, P100 and P115.

### Clinical evaluation of EAU

Mice were anaesthetised and fundi imaged using an otoscope-based fiber-optic light device as described previously (9). In brief, anaesthesia was induced with an intraperitoneal injection of a mixture of 40 mg/kg Vetalar^®^ (Fort Dodge Animal Health Ltd., Southampton, UK) and 0.2 - 1.0 mg/kg Domitor^®^ (Orion Pharma, Espoo, Finland) diluted in injectable water. Pupils were dilated with Minims 1% (w/v) tropicamide, and 2.5% (w/v) phenylephrine hydrochloride (both from Chauvin Pharmaceuticals Ltd, UK). Viscotears Carbomer 2 mg/g liquid gel (Alcon Eyecare UK Ltd., Camberley, UK) was used throughout the procedure as lubrication to prevent corneal drying and lens opacification. Images of the central and peripheral retina were taken. EAU severity was evaluated separately in terms of inflammation and chorioretinal atrophy using a scoring system as previously described (9, 34).

### Histology

Mice (P60) were sacrificed and eyes removed immediately. Eyes were fixed in 2.5% (w/v) glutaraldehyde (Sigma, UK), embedded in resin and processed for standard hematoxylin and eosin (H&E) staining.

### Flow cytometry

Single cell suspensions were prepared from lymph nodes and retinas for flow cytometry analysis. The eye-draining submandibular and skin-draining superficial cervical lymph nodes were collected for specific experiments. Lymph nodes were passed gently through a 70μm cell strainer. Intact, whole retinas were dissected from eye-cups after removing the anterior segment, lens and vitreous. Then retinas were digested in 1 ml PBS containing a final concentration of 10 μg/ml Liberase and 10 μg/ml DNase I (both from Roche, Mannheim, Germany) for 40 min at 37°C. The dissociated lymph node and retina cells were then washed and re-suspended in PBS containing 2% FBS. For the exclusion of dead cells, all samples were stained with Fixable Viability Dye eFluor455 according to manufacturer’s instruction (eBioscience, Hatfield, UK). The cells were then incubated with Fc block CD16/32 (clone 2.4G2) antibody for 10 min at 4°C, followed by surface staining of directly conjugated anti-CD4 (clone GK1.5) APC-Cy7 and anti-CD25 (clone PC61) PE antibodies (both from BD Biosciences, Oxford, UK) for 30 min at 4°C. For the staining of intracellular transcription factors, cells were incubated with Cytofix/Cytoperm (BD Biosciences, Oxford, UK) for 20 min at 4°C followed by staining with directly conjugated antibodies to FoxP3 (clone FJK16S) APC, T-bet (clone 4B10) PE-Cy7 (both from eBioscience, Hatfield, UK) and RORγt PerCp-Cy5.5 (clone Q31-378, BD Biosciences, Oxford, UK) for 30 min at 4°C. For intracellular cytokine staining, dissociated cells were first incubated in RPMI containing 10% FBS, 50ng/ml phorbol 12-myristate-13-acetate, 1μM ionomycin (both from Sigma-Aldrich, St. Louis, MO, USA) and monensin (BD GolgiStopTM, BD Biosciences, UK) for 5h at 5% CO_2_ and 37°C. The cells were then washed and stained with Fixable Viability Dye eFluor455 followed by incubation with Fc-receptor block CD16/32 and then surface staining with CD4 (clone GK1.5) APC-Cy7. Next, BD Cytofix/Cytoperm was used for fixation before staining of intracellular cytokines using directly conjugated anti-mouse IFNγ (clone XMG1.2) APC and IL-17A (clone TC11-18H10) PE (both from BD Biosciences, Oxford, UK). All flow cytometry experiments were acquired using a BD LSRII flow cytometer (BD Bioscience, UK) with collection of least 10^5^ events for each sample. Data analysis was performed using FlowJo, LLC for Windows, version 10 (TreeStar Inc., Ashland, OR, USA).

### Statistical analysis

Data are presented as mean ± SEM. N is indicated in the figure legends. Statistical analysis was performed using GraphPad Prism 9 (La Jolla, CA, USA). For parametric data, unpaired Student’s *t-test* was used to compare between two groups. For non-parametric data, differences between all groups were analysed using Kruskal-Wallis test and Mann-Whitney test was performed between specific groups of interest. Statistical significance was considered when *p*<0.05.

## Results

### Delivery of specific antigen via DEC 205 prevents spontaneous EAU in dTg mice

**Figure 1:**
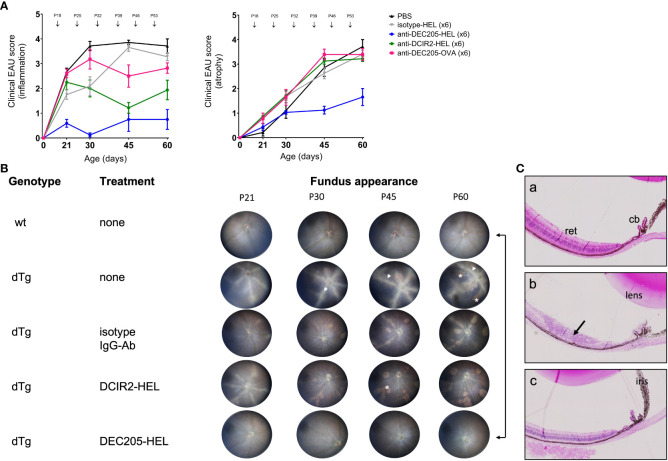
Prevention of Spontaneous Experimental Autoimmune Uveoretinitis (EAU) in HEL-IRBP Double Transgenic mice by Antibody-HEL fusion proteins. **(A)** Dtg HEL/TCR mice were treated with weekly s.c. injection of DEC205-HEL (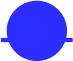), DCIR2-HEL (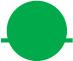), DEC205-OVA (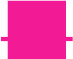), isotype-HEL (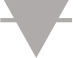) and PBS (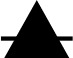) at postnatal (P) days indicated by arrows. Fundus images were acquired at P21, P30, P45, P60 and graded for inflammation and atrophy. Sample size: DEC205-HEL (N=8), DCIR2-HEL (N=8), DEC205-OVA (N=7), isotype-HEL (N=8) and PBS (N=7). Differences between groups were analysed using Mann-Whitney test. **(B)** Representative fundus images of healthy wild type mice, untreated dTg mice and dTg mice treated with weekly inoculation of fusion proteins as in **Figure 1A** and images taken at P21, P30, P45 and P60. Fundus images showing severe vasculitis (arrowhead), and atrophy (asterisk). **(C)** Histology of eyes at P30: (a) non-Tg normal mouse posterior segment; (b) dTg mouse showing severe retinal damage associated with extensive granulomatous inflammation (arrow); (c) dTg mouse treated x2 with DEC205-HEL fusion protein showing normal retinal morphology. cb: ciliary body; ret: retina.

As indicated above, EAU in dTg mice develops spontaneously at eye opening (~P21) and progressively worsens to peak of inflammation (Grade 3-4) at P30 (**Figures 1A, B**). Chorioretinal atrophy accompanies the inflammation but develops more slowly (9, 35, 36). Severe inflammation persists until the entire retina is involved while progressively enlarging areas of chorioretinal atrophy reach a maximum (Grade 3-4) around P60 (**Figure 1A**). A single subcutaneous (s.c.) administration of DEC205-HEL fusion protein at P25 did not delay the progression of EAU compared to DCIR2-HEL, OVA-HEL or Ig isotype-HEL fusion proteins (data not shown). In contrast, repeat weekly doses (beginning P18) of DEC205-HEL by s.c. inoculation, effectively prevented development of EAU, both in terms of inflammation and chorioretinal atrophy through P60, leaving an essentially normal-appearing posterior segment of the eye compared to the isotype-HEL-treated control group (**Figures 1A, B**; p=0.0006 for inflammatory score and p=0.0092 for atrophy score at P60). Histological studies confirmed the anti-inflammatory effect of DEC205-HEL fusion protein: whereas PBS-treated dTg mice showed extensive retinal inflammatory cell infiltration and retinal thinning, with granuloma formation and loss of photoreceptors, dTg mice treated with DEC205-HEL fusion protein retained normal retinal morphology (**Figure 1C**). The isotype-HEL-treated control group also showed inflammatory and atrophic changes although the degree was markedly less severe than in PBS-treated group.

Weekly injections of comparable doses of DCIR2-HEL fusion protein s.c. also suppressed inflammation but were less effective compared to DEC205-HEL and did not prevent chorioretinal atrophy when compared to isotype control-treated mice (**Figure 1A**; p=0.0036 for inflammatory score, p=0.4346 for atrophic score vs isotype control at P60; **Supplementary Figure 1**; **Supplementary Tables 1A, B**). Furthermore, delivery of HEL itself as an Ig isotype fusion protein had no suppressive effects on inflammation (p=0.0834 for Ig isotype fusion protein-treated mice vs PBS-treated mice). Delivery of an irrelevant antigen, OVA, fused to DEC205 antibody had no significant effects on the progression of disease (p=0.1192 for DEC205-OVA treated group vs isotype control) and also had no effect on chorioretinal atrophy which progressed inexorably (p= 0.9525 for DEC-205-OVA vs isotype control).

**Figure 2:**
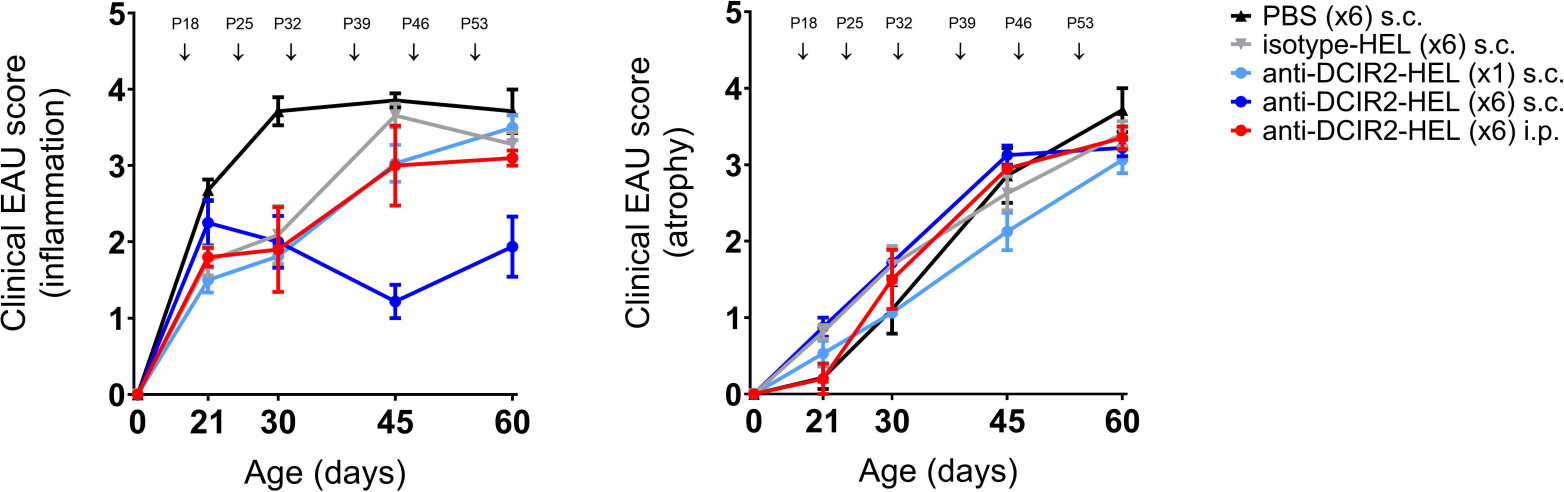
Prevention of spontaneous EAU by s.c. inoculation of DCIR2-HEL fusion protein but not with i.p. administration. Inflammation and atrophy scores of dTg mice treated s.c. with PBS (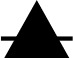), IgG isotype-HEL (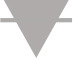), DCIR2-HEL (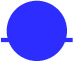) at P18, P25, P32,P39,P46, P53 or with a single s.c. inoculation of DCIR2-HEL (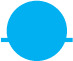) at P18 compared to i.p. administration of DCIR2-HEL fusion protein (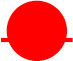) at P18, P25, P32, P39, P46, P53. Weekly administration of DCIR2-HEL s.c. was significantly more efficient in prevention of inflammation compared to i.p delivery (p=0.0167). Sample size: DCIR2-HEL x6 s.c. (N=8), DCIR2-HEL x1 s.c. (N=8), DCIR2-HEL x6 i.p. (N=5), isotype-HEL (N=8) and PBS (N=7). Differences between groups were analysed using Mann-Whitney test.

The DC inhibitory receptor DCIR2 identified by the 33D1 antibody used here is expressed by a subset of tolerogenic splenic DC’s (37) as well as immunogenic cDC2, CXCR1-ve DC (38). Accordingly, we explored whether delivery of this antibody intraperitoneally (i.p.) to target the spleen might enhance its immunosuppressive effect. However, s.c. delivery of DCIR2-HEL fusion protein was more effective in suppressing spontaneous uveitis than i.p. delivery (**Figure 2**: P60 inflammatory score s.c. vs isotype control p= 0.0036; P60 inflammatory score i.p. vs isotype control p= 0.5306). Neither route of administration of DCIR2 fusion protein affected progression of chorioretinal atrophy (P60 atrophic score s.c. vs isotype control p= 0.4346; P60 atrophic score i.p. vs isotype control p= 0.9390).

**Figure 3:**
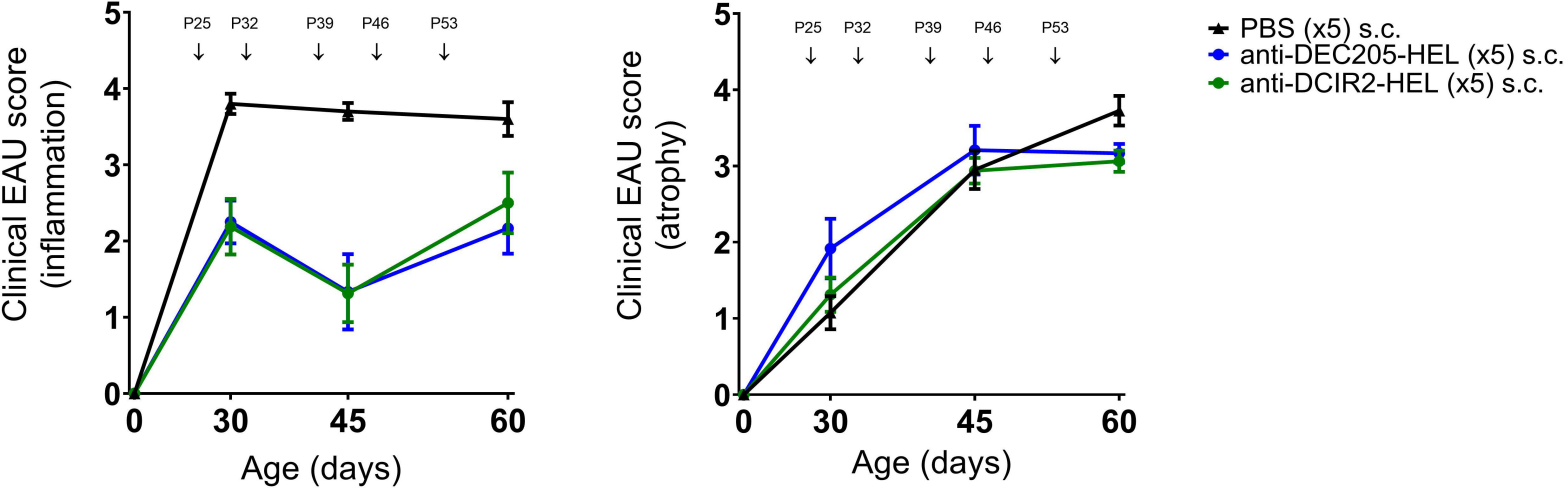
DEC205-HEL and DCIR2-HEL fusion proteins arrest progression of ongoing inflammation but not chorioretinal atrophy. dTg HEL/TCR mice were treated after onset of EAU with weekly s.c. PBS (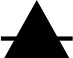), DEC205-HEL (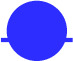) or DCIR2-HEL (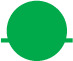) fusion proteins at P25, P32, P39, P46, and P53. Fundoscopy was performed at P30, P45, and P60 for evaluation of EAU progression. Differences between groups were analysed using Mann-Whitney test. DEC205-HEL and DCIR2-HEL fusion protein prevented progression of retinal inflammation (p=0.0061 and p=0.0141 respectively) but despite reaching significantly reduced score in comparison to PBS injected group did not prevent development of atrophy (p=0.0128 and p=0.0042 respectively). Sample size: DEC205-HEL (N=6), DCIR2-HEL (N=8), and PBS (N=10).

**Figure 4:**
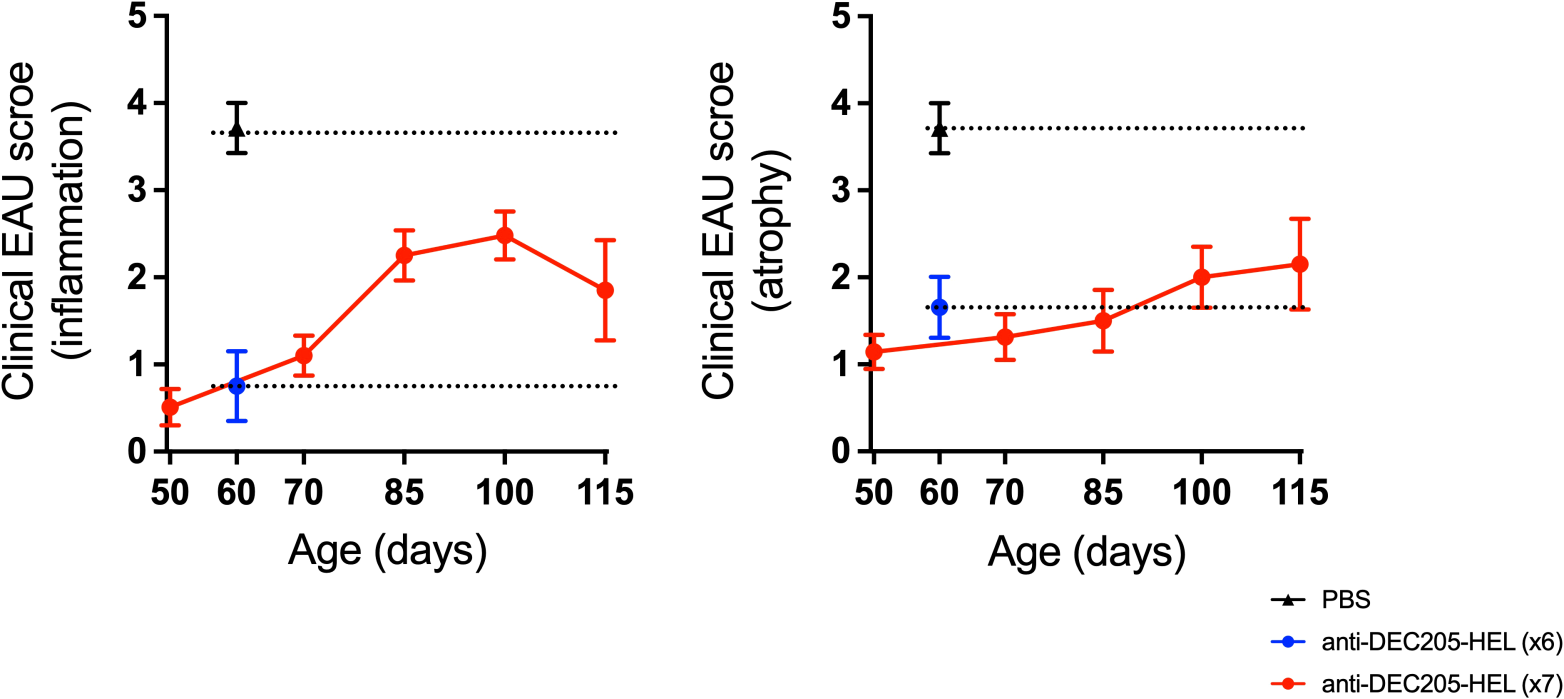
Development of EAU recommenced after cessation of DEC205-HEL fusion protein treatment but was less severe. dTg HEL/TCR mice received repeated weekly treatment of x7 DEC205-HEL fusion protein (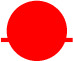) *via* s.c. injection at P18, P25, P32, P39, P46, P53 and P60. Fundoscopy was performed at P50, P70, P85, P100 and P115 for evaluation of retinal inflammation and atrophy. Mice which received weekly treatment of PBS (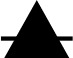) reached grade 4 EAU by P60 (data for P60 time point included for reference). DEC205-HEL treated mice had minimal signs of EAU at P60 (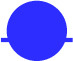) but after stopping treatment beyond P60 began to develop signs of EAU (both inflammation plus atrophy) but until P115 did not reach the level of severity of the PBS treated mice (p=0.0038 for both inflammatory and atrophy). Sample size: P115 DEC205-HEL (N=5), P60 PBS (N=7), P60 DEC205-HEL (N=8). Differences between groups were analysed using Mann-Whitney *U* test.

Administration of weekly doses of DEC205-HEL and DCIR2-HEL fusion proteins, beginning after disease onset (P25), arrested any further development of inflammation (p=0.0061 for DEC205-HEL vs PBS at P60; p=0.0141 for DCIR2-HEL vs PBS at P60) but was only minimally effective in preventing progressive development of chorioretinal atrophy (p=0.0128 for DEC205-HEL vs PBS at P60; p=0.0042 for DCIR2-HEL vs PBS at P60) (**Figure 3**). In addition, cessation of DEC205-HEL treatment after P60 was accompanied by a recurrence of inflammation, but not to the same degree as in PBS-treated control mice (**Figure 4**: p=0.0038 for both inflammatory and atrophic score of P115 DEC205-HEL vs P60 PBS; **Supplementary Table 3**). Furthermore, signs of chorioretinal atrophy began to appear once treatment ceased (**Figure 4**).

### Treatment with DEC 205-HEL fusion protein does not prevent EAU induced by adoptive transfer of HEL-activated CD4^+^ Tg T cells

We have previously shown that CD4^+^ HEL TCR (3A9) T cells, but not CD3 double negative (DN) cells, when adoptively transferred to sTg HEL mice leads to EAU 5d post transfer (9). HEL TCR specific T cells require activation with HEL protein *in vitro* in order to induce EAU by adoptive transfer while non-specific activation with anti-CD3/CD28 antibodies is ineffective (9). In addition, disease severity is less than in spontaneous EAU and is both cell dose-dependent and self-limited.

**Figure 5:**
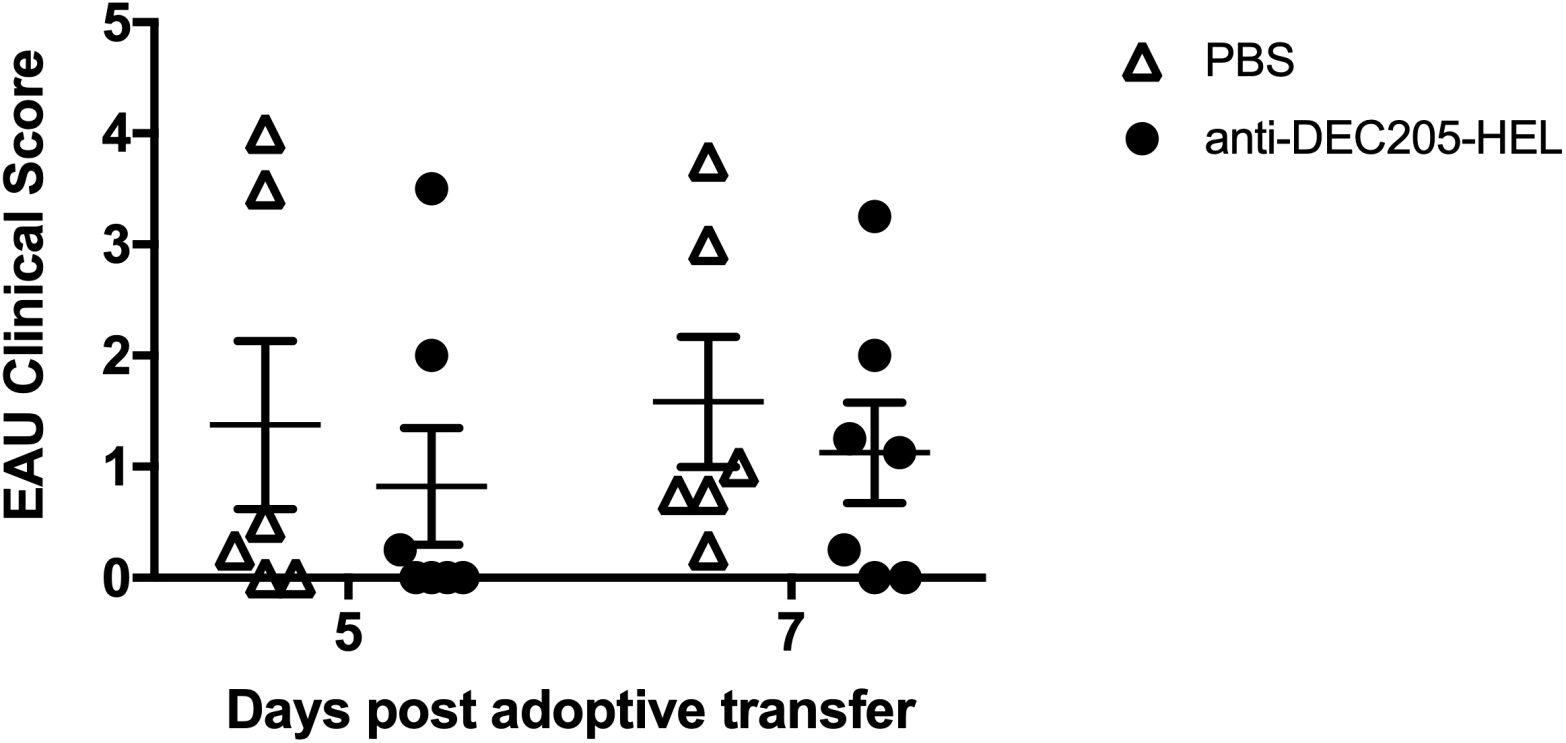
DEC 205-HEL s.c. fusion protein fails to prevent EAU induced by i.v. adoptive transfer of HEL-activated sTg TCR (3A9) lymphocytes to stg HEL mice. Lymph node and spleen cells were processed from sTg TCR (3A9) mice and cultured at 1:1 ratio with HEL protein (1μM) for 72h. sTg HEL mice were treated with s.c. injection of PBS or DEC205-HEL fusion protein at P21, 24h prior to adoptive transfer of 5 x 10^6^ cultured cells via tail vein injection at P22. Fundus images of sTg HEL mice were taken at 5 days (P27) and 7 days (P29) post adoptive transfer for evaluation of EAU severity. There was no difference in EAU inflammatory scores between mice which received PBS and DEC205-HEL fusion protein (p=0.4056 and p=0.6550 for day 5 and 7 post adoptive transfer respectively). Sample size: DEC205-HEL (N=7), PBS (N=6). Differences between groups were analysed using Mann Whitney *U* test.

We wished to determine whether treatment with DEC205-HEL fusion protein would prevent EAU induced by adoptive transfer of *in vitro* HEL-activated T cells. sTg HEL mice were treated s.c. with DEC205-HEL fusion protein or PBS followed 1 day later with i.v. adoptive transfer of 5.0 x 10^6^ HEL TCR (3A9) cells which had been incubated with 1μM HEL protein for 3 days *in vitro*. EAU developed beginning at day 5 post transfer and with 100% incidence by day 7. There was no difference in disease incidence or severity between DEC205-HEL treated mice and PBS treated control mice (**Figure 5**: p=0.4056 and p=0.6550 for DEC205-HEL vs PBS at day 5 and 7 post adoptive transfer respectively).

### Treatment of dTg mice with DEC205-HEL fusion protein prevents infiltration of retinal IFNγ-producing cells and expansion of CD4^+^FoxP3^+^ Tregs in the eye-draining lymph node

We next explored the effect of DEC205-HEL fusion protein on lymphocyte populations in the skin- and eye-draining lymph nodes and in the retina in dTg mice as they developed spontaneous EAU. We have previously shown that dTg mice are profoundly lymphopenic at the onset of EAU (P21) which lessens as the disease progresses but persists for the course of the disease. We have also shown that both IL-17^+^ and INF**γ** ^+^ CD4 T cells as well as CD3^+^ double negative (DN) T cells participate in the development of EAU, the latter population being more prominent in the initial stages of disease (9). Importantly, we have previously shown in mice that the submandibular lymph node (SMLN) is the eye-draining lymph node while the superficial cervical lymph node (SCLN) drains the periocular tissues and cervical skin (39, 40). We used this information to tease out changes in lymphocyte populations in these various sites.

**Figure 6:**
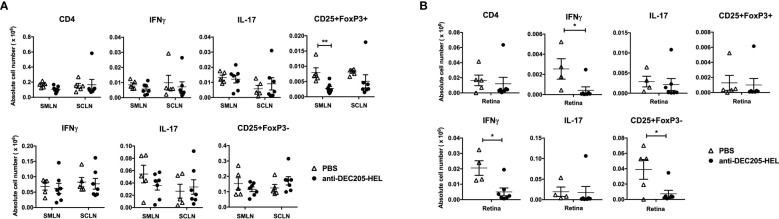
DEC205 fusion protein reduces INFγ^+^ but not IL-17^+^ effector cells in the retina. dTg mice were treated with s.c. injection of DEC205-HEL fusion protein or PBS at P18 and P25 before harvest of the eye draining submandibular lymph node (SMLN), skin draining superficial cervical lymph node (SCLN) and retina at P26. Tissues were processed for cell surface staining of CD4 and CD25, and intracellular staining of IFNγ, IL-17 and FoxP3. **(A)** Lymph node cells and **(B)** retina cells were analysed separately for CD4^+^ (upper panel) and CD4^-^ DN cells (lower panel). **(A)** DEC205-HEL fusion protein treated dTg mice had fewer CD4^+^CD25^+^FoxP3^+^ cells in the SMLN (p=0.0057) and no difference was observed in the CD4^-^DN cells in the DLN between DEC205-HEL fusion protein and PBS treated dTg mice. **(B)** In the retina of DEC205-HEL fusion protein treated mice, both CD4^+^ and CD4^-^DN IFNγ^+^ were markedly reduced as well as CD4DNCD25^+^FoxP3^-^ cells (p=0.0369, p=0.0107 and p=0.0237 respectively) Sample size: DEC205-HEL (N=7), PBS (N=at least 4 per group). Data were analysed using student’s *t*-test, with *p<0.05, **p<0.01 respectively.

We chose one time point (P26) when there would be significant suppression of spontaneous EAU after two doses (P18 and P25) of DEC205-HEL fusion protein (**Figure 1A**). We observed that there was no difference in the absolute numbers of IL-17^+^ or IFNγ^+^ CD4^+^ or DN T cells in either the eye-draining or the skin-draining lymph node **Figure 6A**; **Supplementary Figure 3**). In the retina, DEC205-HEL selectively reduced both the CD4^+^IFNγ^+^ and the DN IFNγ^+^ T cell populations, but had no effect on either IL-17^+^ T cell populations, despite this population being the first cells to infiltrate the retina in this model of EAU (9) (**Figure 6B**). However, there was a significant reduction in DN CD25^+^FoxP3^-^ T cells in the retina, most likely a reflection of the overall markedly suppressed inflammation induced by DEC205-HEL (**Figure 6B**). Interestingly, there was also a significant reduction in CD4^+^CD25^+^FoxP3^+^ T cells in the eye-draining SMLN but not in the skin-draining SCLN although the lack of effect appeared attributable to a single outlier (**Figure 6A**).

**Figure 7:**
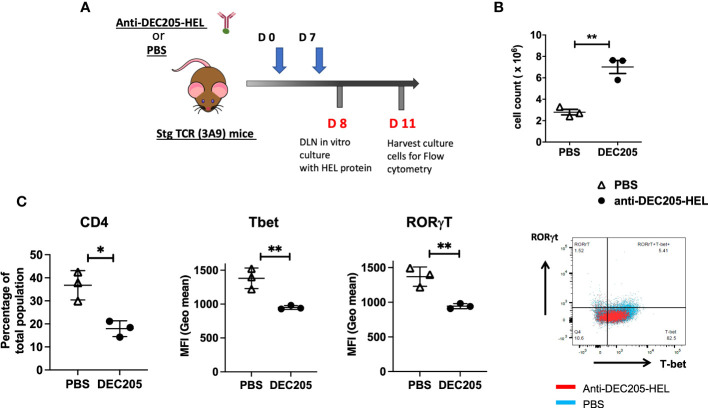
Lymphocytes from sTg DEC205-HEL fusion protein treated mice show reduced T cell activation in response to HEL specific antigen *in vitro*. **(A)** *Experimental design:* Adult (4-6 weeks) sTg TCR (3A9) mice were inoculated with DEC205-HEL fusion protein or PBS s.c. in the neck at day 0 and day 7 of experiment. The skin draining SCLN were harvested at day 8 cultured *in vitro* with 1μM HEL protein for 72h and harvested for flow cytometry of cell surface CD4 and intracellular T-bet and RORγt. **(B)** Absolute cell count of SCLN cells: a significantly greater yield was obtained from DEC205-HEL-treated mice compared to PBS control treated mice at day 8 post inoculation. **(C)** After stimulation *in vitro* with HEL protein, the percentage of CD4^+^T cells was markedly reduced, affecting both T-bet and RORγt expression levels. N=3 mice with *in vitro* cultured cells plated in triplicates and average calculated for each mouse. Data were analysed using student’s *t*-test, with *p<0.05, **p<0.01 respectively.

We further examined the lymphocyte population in the skin-draining SCLN, the site for trafficking steady state DC targeted by inoculations of DEC205-HEL in the cervical skin (**Figure 7A**). Treatment of sTg TCR (3A9) mice with two doses of DEC205-HEL fusion protein (administered s.c. on day 0 and day 7) induced a significantly greater yield in the total number of cells within SCLN on day 8 of the experiment (**Figure 7B**). We then cultured cells isolated from the SCLN for 72h in the presence of HEL protein. Flow cytometric analysis revealed that *in vivo* exposure to DEC205-HEL fusion protein selectively led to a significant reduction in the percentage of the CD4^+^ T cell populations after three days of culture in the presence of HEL, and affected both the Th1 (T-bet) and the Th17 (RORγt) expression amongst CD4^+^ cells (**Figure 7C**).

## Discussion

The HEL : TCR dTg model of EAU is a CD4^+^ T cell mediated disease which develops spontaneously on photoreceptor maturation (~P20/21), and is driven by dysregulation in Teff/Treg cell balance occasioned by profound neonatal lymphopenia (9). Expansion into the lymphocyte space in lymphopenia is known to increase the risk of activating autoreactive T cells (41). The clinical signs in this model have many features resembling idiopathic uncontrolled intraocular inflammation (4) and can be prevented by adoptive transfer of antigen-experienced Tregs (9).

An alternative approach to adoptive Treg transfer in the treatment of inflammatory and autoimmune disease is targeted delivery of specific antigen to steady state DC using antibodies to cell surface proteins fused to the antigen in question. A range of antibodies has been used, targeting both cDC1 and cDC2 with different intended outcomes (17, 18, 21). Delivery of fusion proteins in the context of inflammation (e.g. with an adjuvant), promotes immunity and has been applied to promoting protective immunity against infections and cancer (20, 42–52). In contrast, targeting steady state DC in the absence of additional activation has been used to restore tolerance in autoimmune disease (23, 28, 29, 47, 53, 54).

Different DC surface proteins evoke different responses. For instance, targeting DEC205^+^ cDC1 in the absence of adjuvant is considered a useful strategy for promoting tolerance, while targeting DCIR2^+^ cDC2 may also promote tolerance but is more aligned with promoting immunity (30). Previous studies showed that differential expression of DEC205 and DCIR2 on subsets of DC influenced the outcome of antigen targeting, while monocytic DC (moDC) were significantly less effective in antigen uptake (14, 55). However, the specificity of the target antibody for particular DC subsets is not absolute and the expression of different surface molecules is context-dependent (17, 18).

Data in this report addresses antigen delivery via DC cell surface receptors using specific chimeric antibody fused to HEL in the model of HEL-antigen specific spontaneous EAU described here. Targeting antigen to DEC205 was most effective in preventing development of both the inflammatory disease and the associated chorioretinal atrophy but required early and regular weekly administration. Targeting antigen to DCIR2 was also effective in reducing/preventing inflammation but significantly less so, and was also less effective in preventing chorioretinal atrophy in this model. There was also a small suppressive effect of HEL-fused to IgG but not of irrelevant antigen (OVA) fused to DEC205 antibody, suggesting that at some level targeting proteins to surface receptors on steady state DC using chimeric antibody fusion proteins in the absence of inflammatory stimuli may variably modulate DC function partially by acting via inhibitory Fc receptors.

However, the defining data in this work is the very potent effect of DEC205-HEL fusion protein on suppressing spontaneous EAU. The site of Teff cell activation which initiates disease in this model is unclear but potential sites include the retina, the thymus and the eye-draining lymph node (SMLN) where HEL antigen has been detected in sTg mice (8, 56). Our data show that s.c. cervical skin delivery of DEC205-HEL leads to a marked reduction in both responsiveness of CD4^+^ T cells and expression of T-bet and RORγt after *in vitro* stimulation with HEL, with selective reduction of CD4^+^IFNγ^+^ and DN^-^IFNγ^+^ T cells in the retina *in vivo*. This correlated with a reduction in activated CD25^+^FoxP3^-^ presumed effector T cells in the retina. Inhibition of CD4^+^IFNγ^+^ T cell activity would lead to disease inhibition since, as indicated above, this model is CD4 Th1 type mediated. Interestingly, there was little evidence of induction of Tregs in this model; indeed, there was an apparent reduction of CD4^+^Foxp3^+^ cells in the eye-draining SMLN (**Figure 6**). However, this was probably a reflection of the marked reduction in inflammation (EAU) induced by inhibition of Th1 Teff cells (**Figure 7**). Inflammation in this model is characterised by a significant expansion of Tregs in the retina and the eye-draining lymph node (9) and so the absence of inflammation would be accompanied by an apparent concomitant reduction in Tregs. Interestingly, there was no change in Treg populations in the skin draining SCLN, the site of drainage for migratory DEC205^+^ DCs and selective reduction in CD4^+^ T cell responsiveness to HEL protein following inoculation of DEC205-HEL (**Figure 7C**). This supports a mechanism of action for DEC205-HEL in this model involving inhibition of Th1 Teff cells rather than induction of Tregs. The mechanistic differences between this model and others in which DEC205-antigen fusion proteins mediate their tolerising effect by inducing Tregs, likely relate to the fact that inflammation in this dTg model is lymphopenia-driven in which there is a relative lack of Tregs from the onset of disease (9). An essential role for tolerising DC at the stage of antigen uptake and processing via DEC205-HEL treatment in this model, however, is underscored by the lack of effect of DEC205-HEL in the adoptive transfer model of the disease (**Figure 5**) in which T cell activation is induced by HEL *in vitro* prior to transfer. Furthermore, the results suggest that DC targeting by this method may be ineffective against fully activated T cells. These data conflict with those in an adoptive transfer model of acute colitis (24). However, in the colitis model, Treg, which are a prominent component of the colonic T cell population, appeared to play the greater part in disease control unlike the current model.

It is interesting to speculate on the site of action of DEC205-HEL fusion protein. Inoculation of the fusion protein into the skin will target skin-resident cDC1 as well as DC in the skin-draining SCLN. We have also observed that some soluble DEC205-HEL is likely to enter the circulation via lymph-to-blood transport in DLNs and reach other tissues such as the spleen (data not shown). However, targeting of antigen using DC surface receptor antibody-fusion proteins administered subcutaneously has confirmed local skin cDCs as the site where uptake of antigen for both tolerogenesis (21) and immunogenic vaccination (57) occurs. The life span of such DC as they traffic to the DLN to exert an effect on Teff cells appears to be ~7 days since weekly administration of DEC205-HEL fusion protein which was required to sustain a suppressive effect (**Figure 1A**); moreover, the effect became significantly reduced within days of stopping treatment (**Figure 4**). These data fit well with what is known about the life-span of DC *in vivo* but do not shed light on the site of Teff suppression by DEC205-HEL-treated DC.

Finally, the variable effect of DEC205-HEL and DCIR2-HEL on inflammation and chorioretinal atrophy is noteworthy. Inflammation-associated retinal damage presented as increasingly large areas of atrophy (9), expanding from a perivascular location in the control mice and partially in the DCIR2-HEL treated mice who, unlike the DEC205-HEL-treated mice, always had some degree of inflammation (**Figure 1**). In contrast, when inflammation was completely suppressed as in DEC205-HEL-treated mice the progressive development of atrophic changes suggested they were the result of a residual low grade, albeit ongoing inflammatory response. The role of inflammation in retinal degeneration is now widely recognised.

Overall, the data presented here demonstrate the value of DC targeting in a model of spontaneous autoimmune disease as a means to investigate immunological mechanisms of DC function in restoring immunological tolerance. As a treatment for autoimmune disease, DC targeting may also have value, provided relevant autoantigens can be identified. Currently, clinical trials evaluating tolerogenic DC for the treatment of autoimmune disease and prevention of graft rejection have mainly utilised peripheral blood monocyte’s, cultured in the presence of GM-CSF and IL-4 [reviewed in ref (58)]. However, harvesting rare circulating DC is a laborious and intense process and the possibility of directly targeting cDC1 (and 2) using fusion protein technology for delivery of antigens might be one way forward.

## Data availability statement

The raw data supporting the conclusions of this article will be made available by the authors, without undue reservation.

## Ethics statement

The animal study was approved by University of Aberdeen, Ethical Review Committee, University of Aberdeen, King’s College, Aberdeen AB24 3FX, United Kingdom. The study was conducted in accordance with the local legislation and institutional requirements.

## Author contributions

IPK, TY, RF, KK and LK performed the experiments. RJC generated the transgenic mice and critiqued the paper. CM-G prepared the fusion protein constructs and critiqued the paper. TY, LK, and JVF designed the study. All authors contributed to the article and approved the submitted version.
