# Supplementary material for: Targeted delivery of autoantigen to dendritic cells prevents development of spontaneous uveitis
Source: Front Immunol. 2023 Sep 1;14:1227633. doi: 10.3389/fimmu.2023.1227633 (PMC10505613; doi:10.3389/fimmu.2023.1227633)
Supplement: Supplementary file 1 [file Presentation_1.pptx]

## Slide 1
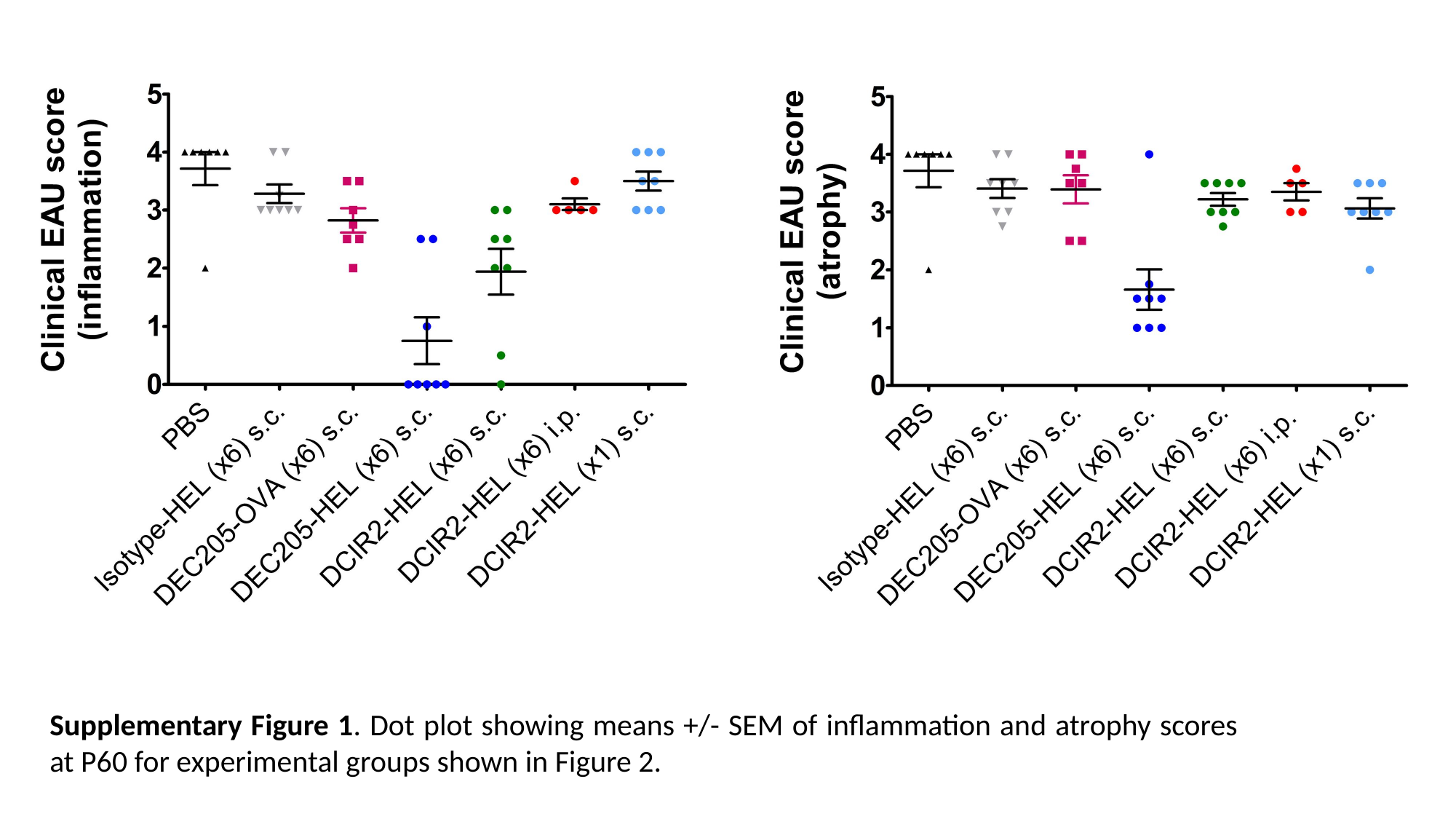

Supplementary Figure 1. Dot plot showing means +/- SEM of inflammation and atrophy scores at P60 for experimental groups shown in Figure 2.

## Slide 2
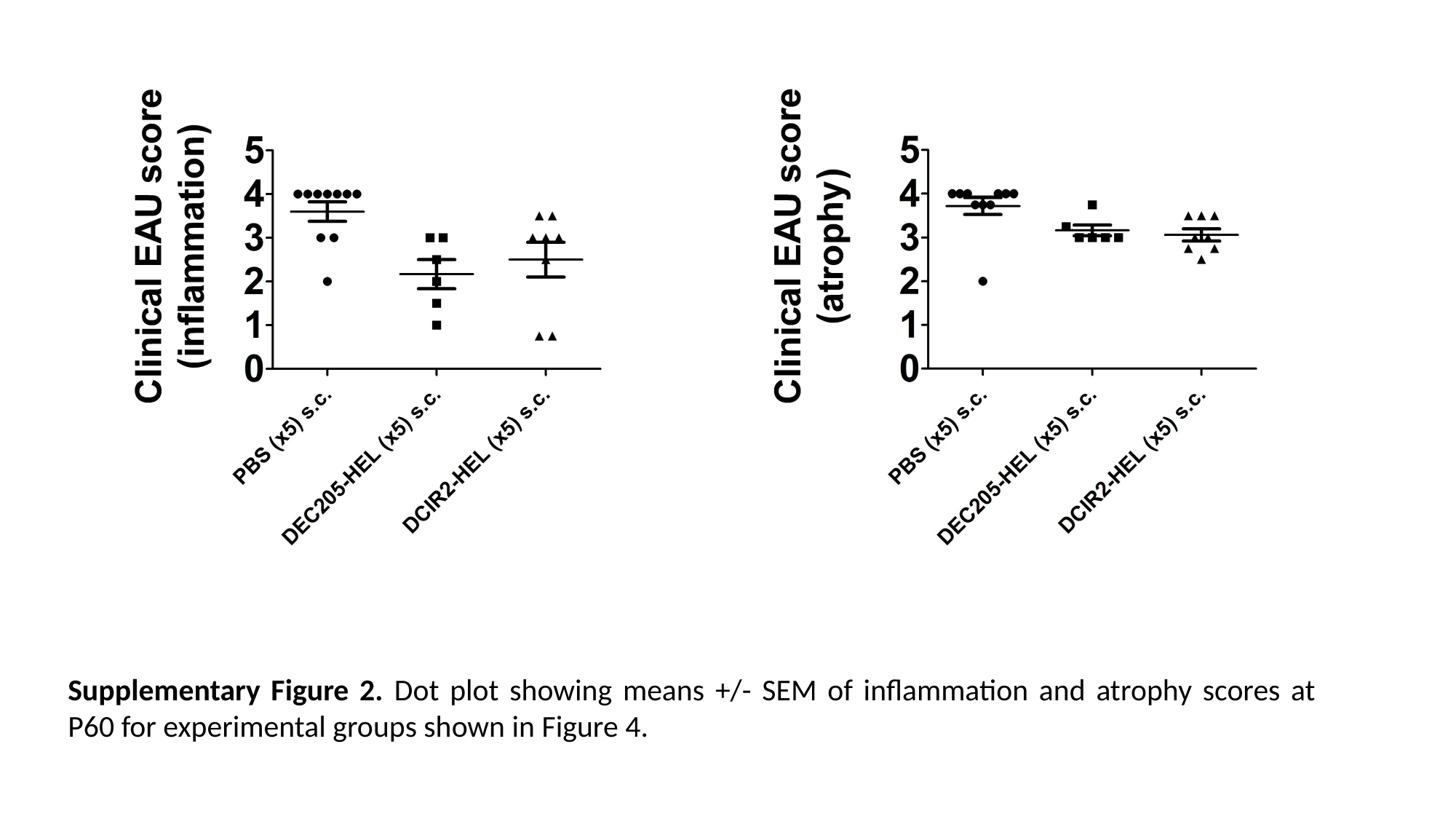

Supplementary Figure 2. Dot plot showing means +/- SEM of inflammation and atrophy scores at P60 for experimental groups shown in Figure 4.

## Slide 3
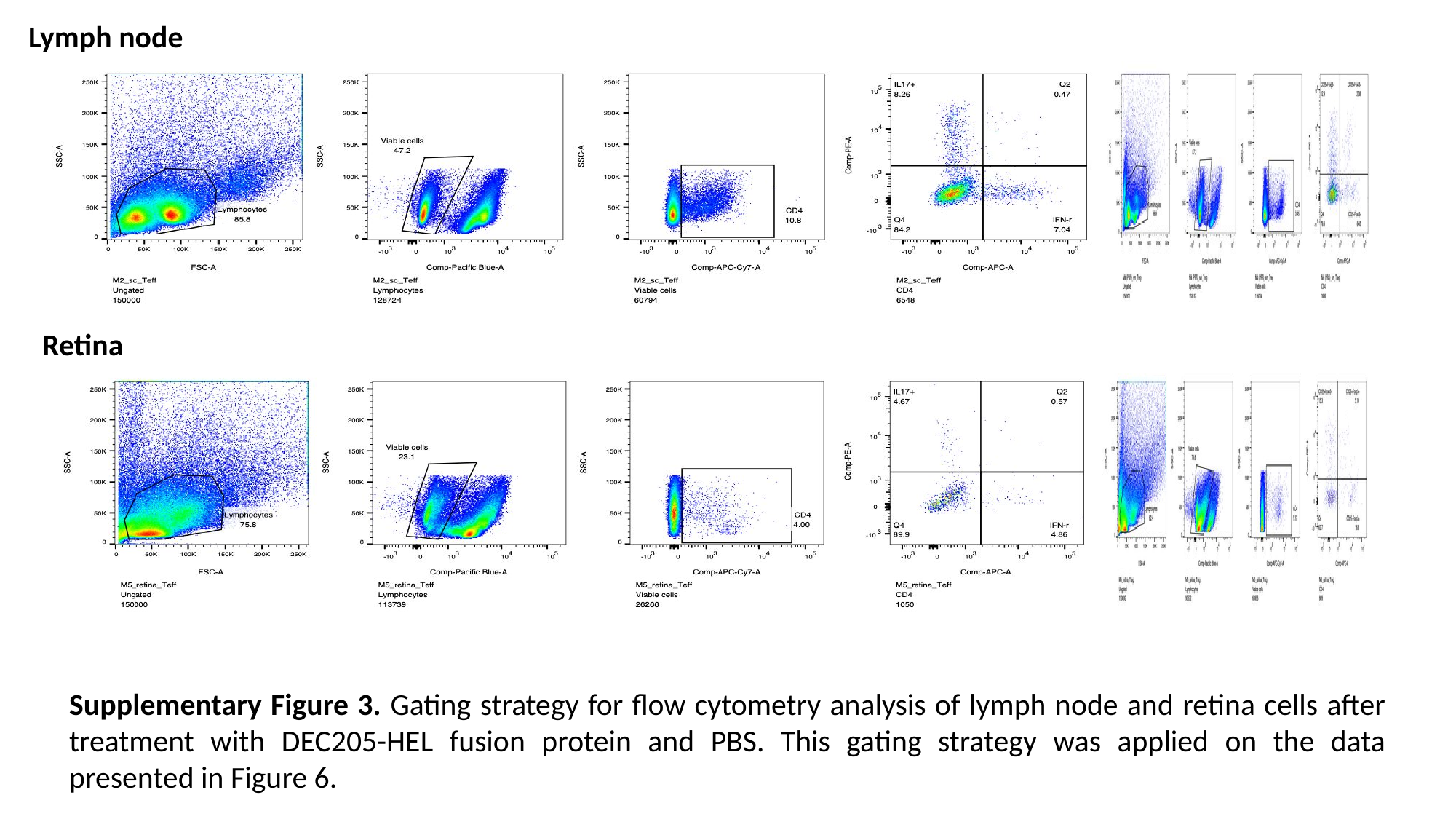

Lymph node
Retina
Supplementary Figure 3. Gating strategy for flow cytometry analysis of lymph node and retina cells after treatment with DEC205-HEL fusion protein and PBS. This gating strategy was applied on the data presented in Figure 6.

## Slide 4
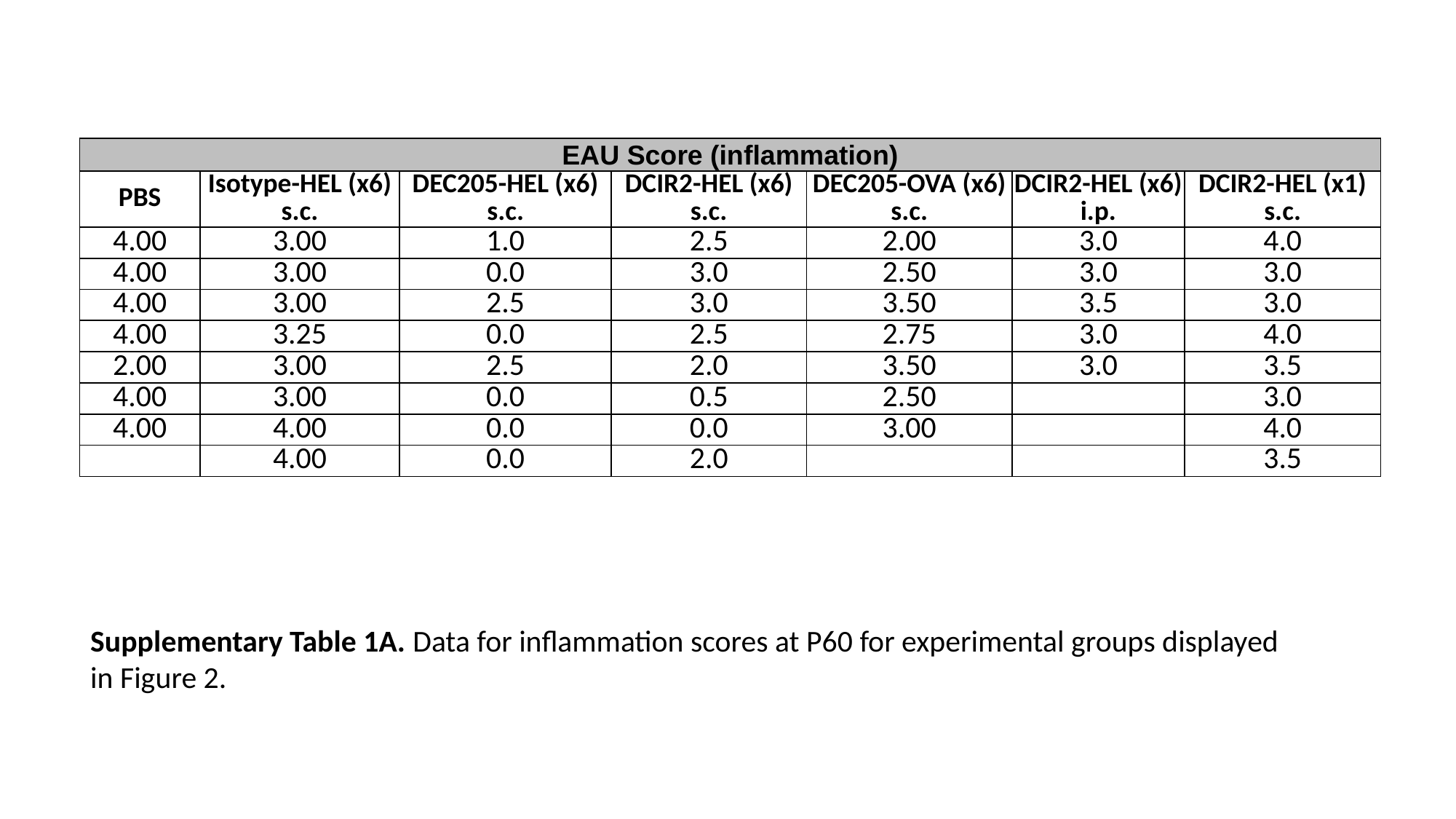

| EAU Score (inflammation) | Isotype-HEL (x6) s.c. | DEC205-HEL (x6) s.c. | DCIR2-HEL (x6) s.c. | DEC205-OVA (x6) s.c. | DCIR2-HEL (x6) i.p. | DCIR2-HEL (x1) s.c. |
| --- | --- | --- | --- | --- | --- | --- |
| PBS | Isotype-HEL (x6) s.c. | DEC205-HEL (x6) s.c. | DCIR2-HEL (x6) s.c. | DEC205-OVA (x6) s.c. | DCIR2-HEL (x6) i.p. | DCIR2-HEL (x1) s.c. |
| 4.00 | 3.00 | 1.0 | 2.5 | 2.00 | 3.0 | 4.0 |
| 4.00 | 3.00 | 0.0 | 3.0 | 2.50 | 3.0 | 3.0 |
| 4.00 | 3.00 | 2.5 | 3.0 | 3.50 | 3.5 | 3.0 |
| 4.00 | 3.25 | 0.0 | 2.5 | 2.75 | 3.0 | 4.0 |
| 2.00 | 3.00 | 2.5 | 2.0 | 3.50 | 3.0 | 3.5 |
| 4.00 | 3.00 | 0.0 | 0.5 | 2.50 | | 3.0 |
| 4.00 | 4.00 | 0.0 | 0.0 | 3.00 | | 4.0 |
| | 4.00 | 0.0 | 2.0 | | | 3.5 |
Supplementary Table 1A. Data for inflammation scores at P60 for experimental groups displayed in Figure 2.

## Slide 5
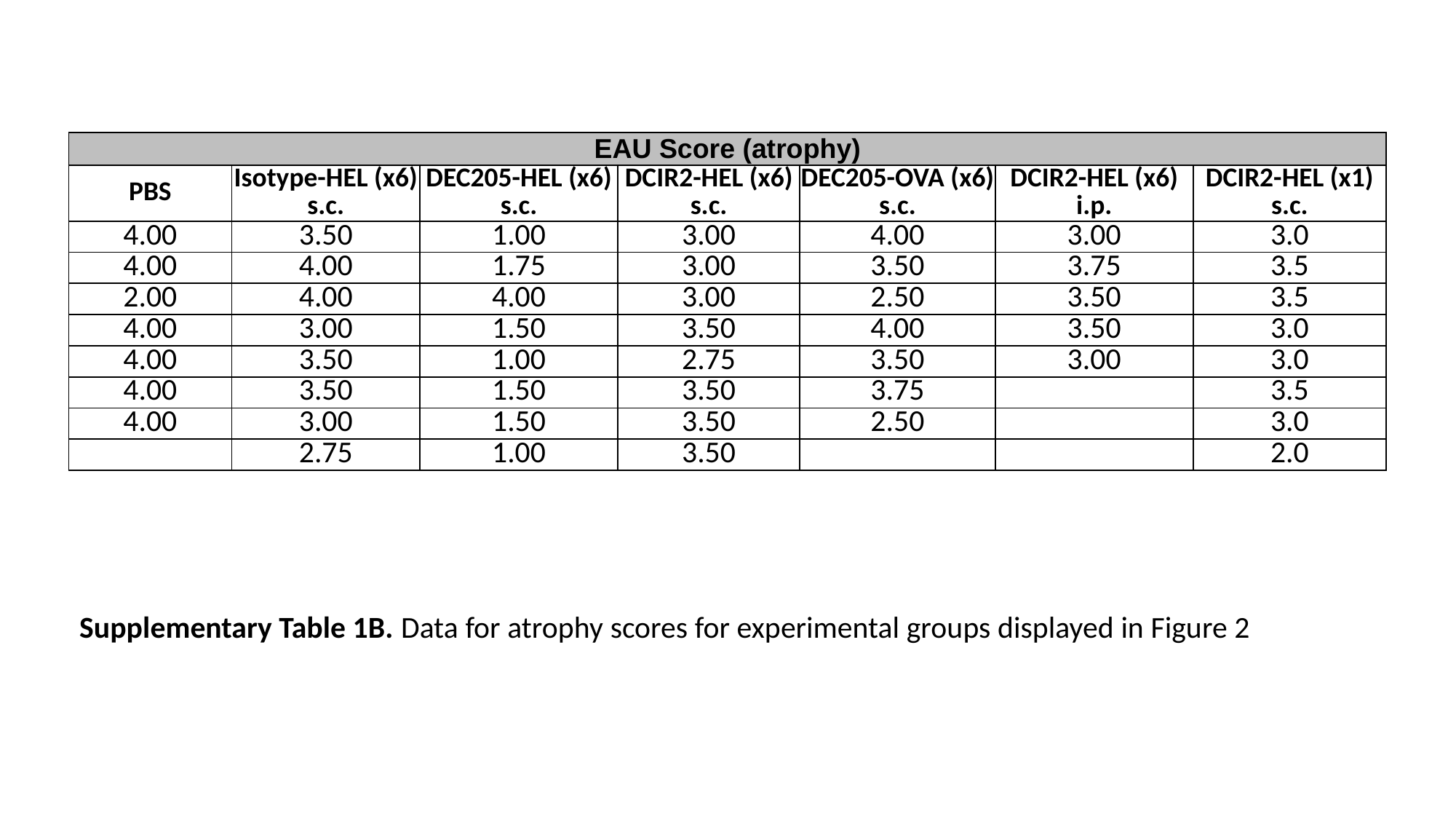

| EAU Score (atrophy) | Isotype-HEL (x6) s.c. | DEC205-HEL (x6) s.c. | DCIR2-HEL (x6) s.c. | DEC205-OVA (x6) s.c. | DCIR2-HEL (x6) i.p. | DCIR2-HEL (x1) s.c. |
| --- | --- | --- | --- | --- | --- | --- |
| PBS | Isotype-HEL (x6) s.c. | DEC205-HEL (x6) s.c. | DCIR2-HEL (x6) s.c. | DEC205-OVA (x6) s.c. | DCIR2-HEL (x6) i.p. | DCIR2-HEL (x1) s.c. |
| 4.00 | 3.50 | 1.00 | 3.00 | 4.00 | 3.00 | 3.0 |
| 4.00 | 4.00 | 1.75 | 3.00 | 3.50 | 3.75 | 3.5 |
| 2.00 | 4.00 | 4.00 | 3.00 | 2.50 | 3.50 | 3.5 |
| 4.00 | 3.00 | 1.50 | 3.50 | 4.00 | 3.50 | 3.0 |
| 4.00 | 3.50 | 1.00 | 2.75 | 3.50 | 3.00 | 3.0 |
| 4.00 | 3.50 | 1.50 | 3.50 | 3.75 | | 3.5 |
| 4.00 | 3.00 | 1.50 | 3.50 | 2.50 | | 3.0 |
| | 2.75 | 1.00 | 3.50 | | | 2.0 |
Supplementary Table 1B. Data for atrophy scores for experimental groups displayed in Figure 2

## Slide 6
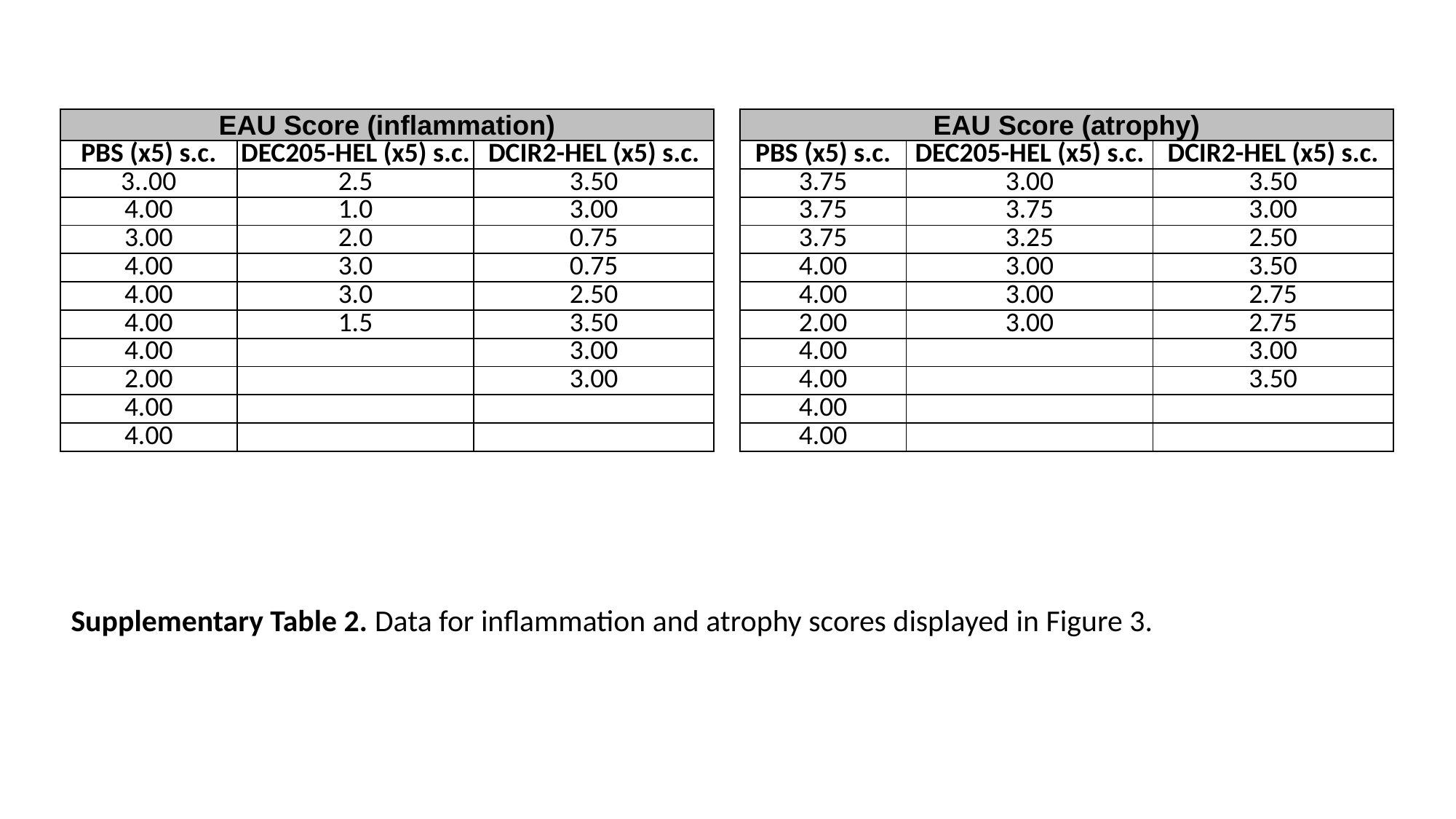

| EAU Score (inflammation) | | |
| --- | --- | --- |
| PBS (x5) s.c. | DEC205-HEL (x5) s.c. | DCIR2-HEL (x5) s.c. |
| 3..00 | 2.5 | 3.50 |
| 4.00 | 1.0 | 3.00 |
| 3.00 | 2.0 | 0.75 |
| 4.00 | 3.0 | 0.75 |
| 4.00 | 3.0 | 2.50 |
| 4.00 | 1.5 | 3.50 |
| 4.00 | | 3.00 |
| 2.00 | | 3.00 |
| 4.00 | | |
| 4.00 | | |
| EAU Score (atrophy) | DEC205-HEL (x5) s.c. | DCIR2-HEL (x5) s.c. |
| --- | --- | --- |
| PBS (x5) s.c. | DEC205-HEL (x5) s.c. | DCIR2-HEL (x5) s.c. |
| 3.75 | 3.00 | 3.50 |
| 3.75 | 3.75 | 3.00 |
| 3.75 | 3.25 | 2.50 |
| 4.00 | 3.00 | 3.50 |
| 4.00 | 3.00 | 2.75 |
| 2.00 | 3.00 | 2.75 |
| 4.00 | | 3.00 |
| 4.00 | | 3.50 |
| 4.00 | | |
| 4.00 | | |
Supplementary Table 2. Data for inflammation and atrophy scores displayed in Figure 3.

## Slide 7
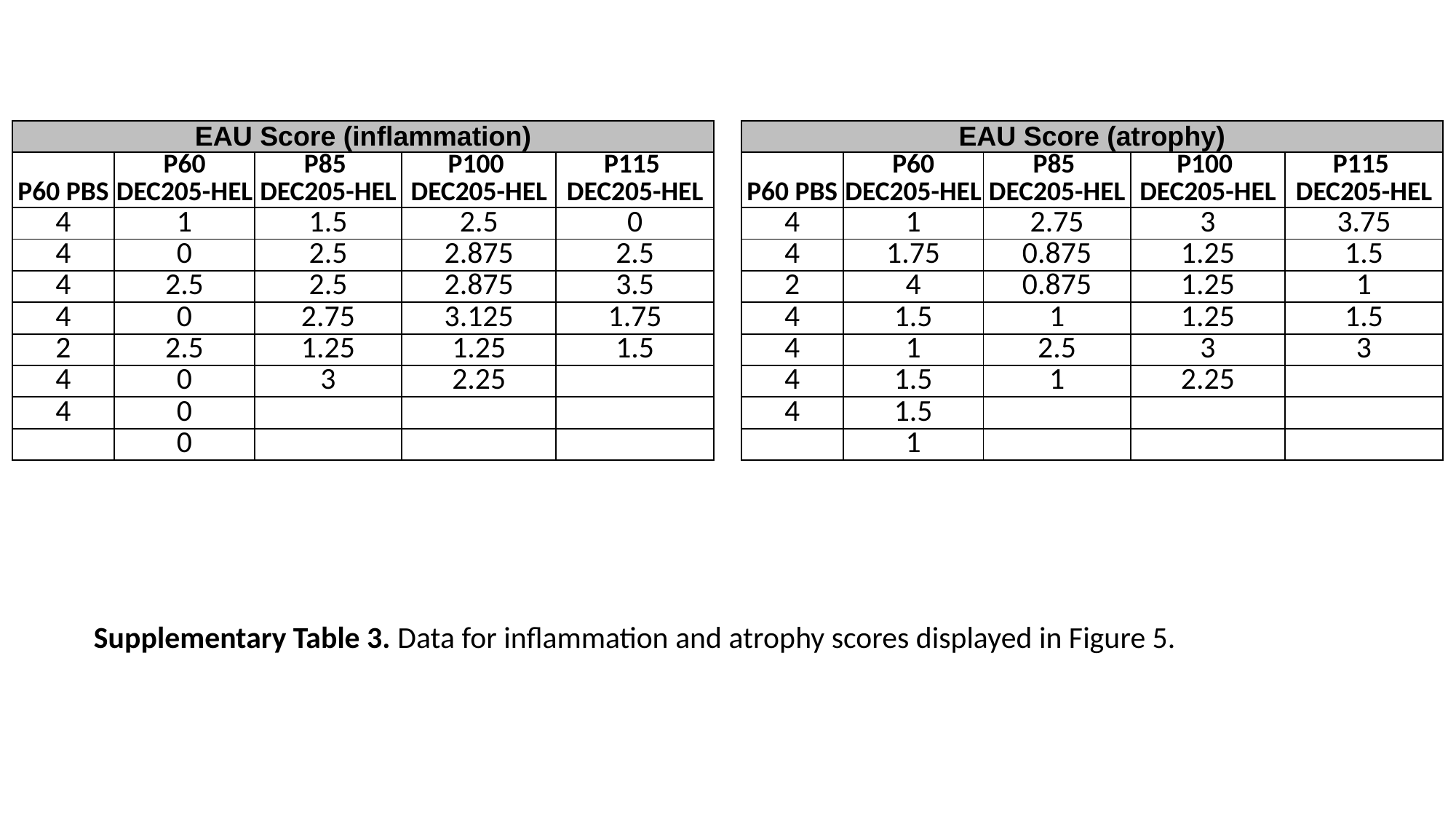

| EAU Score (inflammation) | | | | |
| --- | --- | --- | --- | --- |
| P60 PBS | P60 DEC205-HEL | P85 DEC205-HEL | P100 DEC205-HEL | P115 DEC205-HEL |
| 4 | 1 | 1.5 | 2.5 | 0 |
| 4 | 0 | 2.5 | 2.875 | 2.5 |
| 4 | 2.5 | 2.5 | 2.875 | 3.5 |
| 4 | 0 | 2.75 | 3.125 | 1.75 |
| 2 | 2.5 | 1.25 | 1.25 | 1.5 |
| 4 | 0 | 3 | 2.25 | |
| 4 | 0 | | | |
| | 0 | | | |
| EAU Score (atrophy) | | | | |
| --- | --- | --- | --- | --- |
| P60 PBS | P60 DEC205-HEL | P85 DEC205-HEL | P100 DEC205-HEL | P115 DEC205-HEL |
| 4 | 1 | 2.75 | 3 | 3.75 |
| 4 | 1.75 | 0.875 | 1.25 | 1.5 |
| 2 | 4 | 0.875 | 1.25 | 1 |
| 4 | 1.5 | 1 | 1.25 | 1.5 |
| 4 | 1 | 2.5 | 3 | 3 |
| 4 | 1.5 | 1 | 2.25 | |
| 4 | 1.5 | | | |
| | 1 | | | |
Supplementary Table 3. Data for inflammation and atrophy scores displayed in Figure 5.
